# Evaluation of the Applicability of a 3D-Printed Inert Minitablet Core as a Carrier for Modified-Release Drug Delivery System

**DOI:** 10.3390/pharmaceutics18030295

**Published:** 2026-02-27

**Authors:** Ádám Tibor Barna, Christian Fleck, Adrienn Katalin Demeter, Bence Borbás, Bálint Basa, Emese Balogh, Réka Angi, Nikolett Kállai-Szabó, István Antal

**Affiliations:** 1 Department of Pharmaceutics, Semmelweis University, Hőgyes E. Street 7-9, 1092 Budapest, Hungary; barna.adam@semmelweis.hu (Á.T.B.); christian.petszulat@stud.semmelweis.hu (C.F.); demeter.adrienn.katalin@semmelweis.hu (A.K.D.); borbas.bence@semmelweis.hu (B.B.); basa.balint@semmelweis.hu (B.B.); balogh.emese@semmelweis.hu (E.B.); kallai.nikolett@semmelweis.hu (N.K.-S.); 2 Center for Pharmacology and Drug Research & Development, Semmelweis University, Üllői Street 26, 1085 Budapest, Hungary; angi.erzsebet@semmelweis.hu; 3 Department of Pharmaceutical Chemistry, Semmelweis University, Hőgyes E. Street 9, 1092 Budapest, Hungary

**Keywords:** 3D printing, inert minitablet cores, minitablets, multi-unit particulate systems (MUPS), drug release, controlled-release coating, 3D-printed subunit

## Abstract

**Background/Objectives**: The growing demand for personalised, patient-centric drug delivery systems has driven innovation in pharmaceutical manufacturing, particularly in multi-unit particulate systems (MUPS). **Methods**: In this study, inert cores with tailor-made geometry for multi-particulate formulations were fabricated with high-resolution stereolithography (SLA) 3D printing. By a printable photopolymer resin, dimensionally accurate and mechanically robust starter cores were produced. The additively manufactured inert subunits were drug-layered with ibuprofen sodium using a fluidised bed process. Then, a controlled-release film coating of Eudragit RS 30D was applied with varying coating thicknesses. The initial 3D-printed subunits, together with the drug-layered and finally film-coated microparticles, were characterised by image analysis, Raman microspectroscopic measurements, and official methods of the European Pharmacopoeia. **Results**: The combined approach of 3D printing and traditional pharmaceutical processing proved highly effective. The 3D-printed cores demonstrated both flexibility in design and consistency in performance. **Conclusions**: These findings highlight the feasibility of using 3D printing to produce patient-specific, functional cores in multi-particulate systems that can be easily modified according to the patient’s needs. The fabricated minitablets can be used as alternatives to widely used inert cores. Integrating additive manufacturing with conventional coating techniques offers promising new avenues for developing next-generation, personalised drug delivery solutions.

## 1. Introduction

Oral administration continues to be the most widely used route of drug delivery [1]. Compared with conventional single-unit dosage forms, multiparticulate systems administered orally offer several therapeutic and pharmaceutical advantages [2]. Dosage forms composed of multiple micron-sized subunits enable a range of therapeutic strategies, including the simultaneous administration of loading and maintenance doses, the combination of otherwise incompatible active pharmaceutical ingredients within a single dosage form, and improved flexibility in dosing. Additionally, modified-release tablets can be divided, and capsules can be opened without compromising their performance [3]. The ability to subdivide the dosage form is particularly beneficial for patients experiencing swallowing difficulties [4].

Multiparticulate systems can be constructed from a wide variety of building blocks [5]. Pellets are among the most commonly used subunits and are typically characterised by either homogeneous or heterogeneous structures, most often designed for modified drug release [6]. Minitablets (also referred to as microtablets, microtabs, minitabs, or mini-tablets) represent another regularly shaped particulate system and have been considered an alternative to spherical particles since the 1960s [7]. One of their key advantages over pellets is their well-defined and uniform geometry, which allows for more accurate surface area calculations, whereas conventional industrial pellets are spherical but vary in size even within a single batch [8]. This, in turn, enables a more precise determination of the required amount of film-forming polymer and reduces variability between individual units [9]. Tablet-shaped micro particles are already used in commercially available medicinal products, even though minitablets are still not explicitly defined as a separate dosage form in current pharmacopoeias [10].

Lura et al. classified minitablets based on their functional properties, distinguishing between orally disintegrating (ODMT, orodispersible mini-tablet), dispersible, conventional, and modified-release minitablets. In addition to uncoated matrix-based systems, coated minitablets are also widely investigated, where the coating may serve purposes such as taste masking or controlled drug release [11]. Minitablets containing various active pharmaceutical ingredients with homogeneous drug distribution have been reported by several research groups. Various manufacturing approaches are available for producing drug-containing micro-sized tablet dosage forms. Direct compression and hot-melt extrusion (HME) technologies are commonly applied; moreover, Poller demonstrated the feasibility of electrospinning for the preparation of PVP-based, drug-loaded microtablets exhibiting a homogeneous matrix structure [12,13,14]. Similarly, minitablets with comparable internal architectures, characterised by a uniform distribution of the active pharmaceutical ingredient and excipients, have been produced by several research groups using 3D printing.

Krause et al. prepared minitablets from drug-polymer filaments using FDM-based 3D printing [15,16]. The same printing technique was employed by Kim et al., who produced polyhedral-shaped minitablets [17]. Hu et al. developed orally disintegrating minitablets using SSE 3D printing technology [18]. Going one step further, Oldfield demonstrated the fabrication of the entire dosage unit by dual-extrusion 3D printing. In this approach, a drug-loaded filament and a filament consisting solely of excipients were combined to produce a complete multiparticulate dosage form in which the individual subunits were minitablets [19].

These achievements show that versatile 3D printing technologies can be applied for the production of API-containing formulations [20]. The widely used FDM printing combined with the extrusion of customised filament is a promising method in the development of pharmaceutical treatments [21]. From another perspective, the utilization of liquid starting materials is also desirable, which justifies the exploration of the use of SLA printing as well [15]. Placing numerous units onto one printing bed can enhance production and decrease the time needed to treat one or more patients at one dose. However, the previously discussed matrix-production methods should be reconsidered, since if there are any solubility difficulties, a formulation of suspension is inevitable, and the lack of effective mixing procedures leads to an inhomogeneous printed structure and insufficient API content homogeneity [22]. In accordance with these considerations, the formulation of inert cores tends to be a reasonable alternative.

In conventional multiarticulate systems, commercially available sugar-, MCC-, tartaric acid- or isomalt-based starter cores are widely used due to their well-established manufacturability and regulatory acceptance [23,24]. The use of inert cores facilitates the work of pharmaceutical developers and researchers, as the system can be readily adapted to different active pharmaceutical ingredients and dosage strengths through conventional layering processes [25]. However, the geometry, internal structure, and size distribution of such cores are largely predefined and only limitedly adjustable. In contrast, SLA-based fabrication enables precise control over particle geometry and internal architecture at the design stage, offering a level of structural customisation that cannot be achieved with conventional starter pellets. Furthermore, most previously reported 3D-printed minitablets produced by FDM, SSE, or DLP technologies rely on matrix-type formulations in which the active pharmaceutical ingredient is incorporated directly into the printed structure. While these systems demonstrate the feasibility of additive manufacturing, they may limit formulation flexibility and post-processing adaptability.

The objective of the present study was to produce inert minitablet cores that can be used as alternatives to inert cores currently used in drug formulations from printable polymers using SLA 3D printing. After a comprehensive characterization of the printed minitablets, the tablet-shaped micro particles were used as substrates for drug layering via a fluidised-bed process, followed by the application of a film coating to the drug-loaded units. In addition to evaluating drug release behaviour, the reconstitution of the 3D-printed dosage forms was also investigated.

## 2. Materials and Methods

### 2.1. Materials

FLEX80 Transparent Clear Prusament Resin (Prusa Research a.s., Prague, Czech Republic) was used as a 3D-printable photopolymer resin during the additive manufacturing of the inert minitablet cores [26,27,28]. During the post-curing phase, the printlets were washed with isopropyl alcohol (Molar Chemicals Kft., Halásztelek, Hungary). Hydroxypropyl methylcellulose (HPMC; Pharmacoat 606, Shin-Etsu Chemical Ltd., Tokyo, Japan) was used as the binder excipient during the drug layering [29,30]. Sunset Yellow FCF (303CC Sensient Food Colors UK Ltd., Norfolk, UK) was used as the colouring agent for the drug-containing layer. The active ingredient of the model was ibuprofen sodium (lot number: BCCK2792, Sigma-Aldrich Chemie GmbH, Darmstadt, Germany) [31]. Eudragit RS 30D (Evonik Industries AG, Essen, Germany) was used to formulate the release-modifying coating [32]. Micronised talc powder (Sigma-Aldrich Chemie GmbH, Darmstadt, Germany) was added to the film-coating dispersion to enhance its adhesive properties. Triethyl citrate (Sigma-Aldrich Chemie GmbH, Darmstadt, Germany) was used as a plasticiser for the film coating.

### 2.2. Manufacturing

#### 2.2.1. Design

The 3D objects were designed using Autodesk Fusion 360 (Autodesk Inc., San Rafael, CA, USA) [33,34], and the finalised designs were exported as stereolithography files. The diameter of the inert core model was 3.0 mm, and the height of the tablets was set to 2.5 mm [35]. The cylinder had a diameter of 1.5 mm, and 0.5 mm lofting was applied to both horizontal sides, with a point tangent setting used to form the final shape. During the slicing process, the Prusa Slicer (version 2.4.2) (Prusa Research a.s., Prague, Czech Republic) software was utilised to divide the object into multiple well- defined horizontal slices and to define the printing parameters [36]. The exported file was placed on the printing platform, which contained various models to maximise the production capacity.

#### 2.2.2. SLA Printing

**Figure 1:**
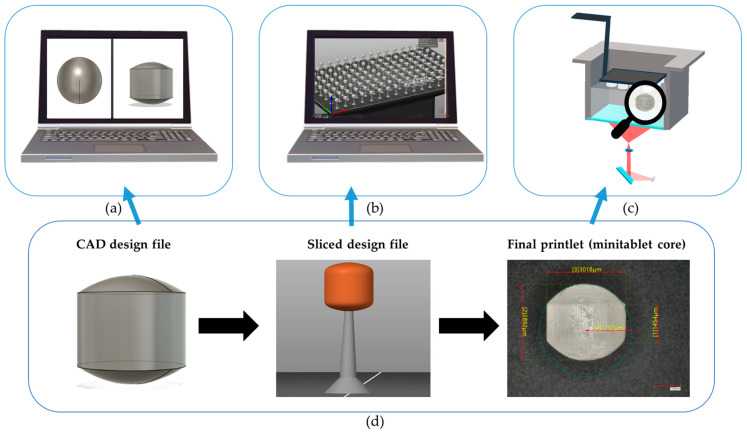
Workflow from the design to the actual printing. (**a**) Computer-Aided Design (CAD), (**b**) Sliced file, (**c**) Final printed inert core, (**d**) 3D printing workflow.

The minitablets were printed using the Original Prusa SL1S SPEED SLA 3D printer (Prusa Research a.s., Prague, Czech Republic) [37]. The objects were additionally manufactured with a layer thickness of 0.025 mm per layer, with the supports beneath the structures. The exposure time was 10 s for the first three layers and 2 s for the subsequent layers. Figure 1 summarizes the workflow from the design to the actual printing [38].

After printing, the structures were cleaned and post-cured using the Original Prusa Curing and Washing Machine (Prusa Research a.s., Prague, Czech Republic) [33]. The printed models were immersed in 2.5 L isopropyl alcohol bath for 5 min, followed by 3 min of drying and an additional 3 min of UV post-curing.

#### 2.2.3. Drug Layering on Inert Minitablet Cores

Drug layering on inert 3D-printed minitablets cores was carried out using the fluidised bed bottom-spray method in an Aeromatic STREA-I laboratory fluidised bed system (Aeromatic-Fielder AG, Bubendorf, Switzerland) [39].

The active ingredient (5.000; *w*/*w*%) and the colouring agent (0.100 *w*/*w*%) were dissolved in a previously prepared (2.000; *w*/*w*%) aqueous hydroxypropyl methylcellulose liquid [40]. Table 1 shows the manufacturing parameters. The weight ratio of the applied dry matter (API, HPMC) to the minitablet core was 1:1.

#### 2.2.4. Film Coating

The additively manufactured minitablets were coated using the previously mentioned fluidised bed coating system with the bottom-spray method [41]. The process parameters are given in Table 1.

Table 2 presents the precise composition of the dispersion employed for modified drug release. This formulation follows the manufacturer’s recommendations and has been utilised in several previous studies [29,30].

In the case of film coatings, samples were taken after 10% and 20% weight increases, with the endpoint considered to be a 30% weight increase.

### 2.3. Physical Characterisation of the Minitablets

#### 2.3.1. Mass Uniformity, Friability, and Hardness

In the European Pharmacopoeia (Ph. Eur.), 3D printlet requirements are not currently determined because of the novelty of the method. Therefore, tests for mass uniformity, friability, and hardness were conducted according to the procedures outlined in Chapter 2.9.5 (Uniformity of Mass of Single-Dose Preparations), Chapter 2.9.7 (Friability of Uncoated Tablets) and 2.9.8 (Resistance to crushing of tablets) of the 11th European Pharmacopoeia [42]. The mass uniformity tests used an analytical balance (*n* = 20; Sartorius LA 230S, Sartorius AG, Göttingen, Germany). A tablet friability apparatus (Erweka TAR 120, Langen, Germany) was employed to determine friability, and mechanical strength was evaluated with a tablet hardness tester (*n* = 20; Erweka TBH 200 TD type, Erweka AR, Langen, Germany) [43,44]. Average values were recorded for each parameter tested.

#### 2.3.2. Image Analyses

The images of the particles (*n* = 75) were made using a Keyence VHX-7000 optical microscope (Keyence Corp., Osaka, Japan). This advanced imaging system played a crucial role in capturing high-resolution images of the particles, enabling the measurement of detailed visual inspections of various physical attributes, such as size, shape, and surface morphology [36]. ImageJ (version 1.54f) software was used to determine these parameters.

#### 2.3.3. Raman Microscopic Analysis

The 3D-printed minitablet was trimmed using a Leica EM RAPID (Leica Microsystems GmbH, Wetzlar, Germany) milling system. Approximately 1700 µm was removed from the surface to expose the internal structure [45]. The resulting flat cross-section was used for Raman mapping without further polishing.

A Thermo Fisher DXR dispersive Raman spectrometer (Thermo Fisher Scientific Inc., Waltham, MA, USA) equipped with a CCD camera and a 780 nm diode laser was used to acquire both individual Raman spectra and chemical maps [46]. A microscopic lens with 10× magnification was used. Measurements were carried out at a laser power of 24 mW, using a 50 µm slit size, an exposure time of 20 s, and five accumulations per spectrum. Background correction was based on 512 scans, and the spectral acquisition range was 3200–50 cm^−1^. Cosmic ray and fluorescence corrections were applied during acquisition.

Raman chemical mapping was conducted on an area of 946 µm × 947 µm with a step size of 22 µm, allowing for the spatial distribution of individual components to be visualised using spectral profiling. For the presentation of the individual spectra, only a constant vertical offset along the y-axis was applied to improve visibility; no baseline correction, smoothing or other post-processing was performed. The Raman spectra were normalised to minimise intensity variations between different regions of the sample. Data evaluation was performed using OMNIC (version 8.1.0) software [47].

#### 2.3.4. Study of Drug Release

In vitro dissolution tests were conducted at a Hanson SR-8 Plus™ Dissolution Test Station (Hanson Research, Los Angeles, CA, USA) employing the rotating basket method (USP Apparatus I) at a rotation speed of 100 rpm [34]. The volume of the medium was 900 mL at pH 6.8. During measurement, it was tempered to 37.0 ± 0.2 °C. 5 mL samples were taken automatically at specified time points through 10 µm pore size membrane full-flow filters using the Hanson^®^ AutoPlus Multifill collector (Hanson Research, Los Angeles, CA, USA). After each sampling, the medium was replaced with 5 mL of fresh buffer solution. Six parallel measurements were performed on each sample.

The concentrations of ibuprofen sodium in the dissolution samples were determined with UV spectroscopy with an Agilent 8453 UV–VIS spectrophotometer (Agilent Technologies, Waldbronn, Germany) at a wavelength of 222 nm [48].

#### 2.3.5. Kinetic Analysis of Dissolution Data

To study the mechanism of drug dissolution from the ibuprofen sodium-loaded film-coated minitablets, the release data were fitted to various release kinetic models. To account for the observed lag time (*t_lag_*), kinetic modelling was performed using a time-shifted approach, where the effective time was defined as (*t* − *t_lag_*). The Higuchi (Equation (1)), and Korsmeyer-Peppas (Equation (2)) models were fitted to the release data for *t* > *t_lag_*. The Korsmeyer-Peppas model was applied to the initial 65% of drug release. The equations of the models are as follows:

(1) $$\mathrm{Higuchi}:\;M_{t}=k_{H}\sqrt{t-t_{lag}}$$

(2) $$\mathrm{Korsmeyer-Peppas}:\;\frac{M_{t}}{M_{\infty }}=k_{KP}{\left(t-t_{lag}\right)}^{n}$$

where *M_t_* is the amount of drug released at time *t*, *M*_∞_ is the total drug released at infinite time, *k_H_* and *k_KP_* are the release rate constants, and *n* is the diffusional exponent [49,50]. The goodness of fit for both the Higuchi and Korsmeyer-Peppas models was assessed using the coefficient of determination (*R*^2^) (Equation (3)), calculated as follows:

(3) $$R^{2}=1-\frac{SS_{res}}{SS_{tot}}\;$$

where *SS_res_* and *SS_tot_* are the residual and total sum of squares, respectively.

## 3. Results

### 3.1. Manufacturing Outcomes

**Figure 2:**
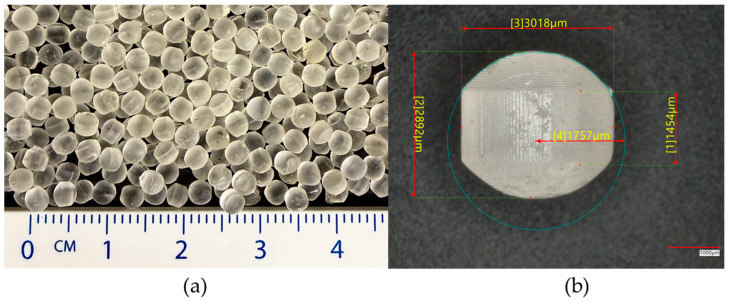
Images of printlets; (**a**) 3D-printed inert minitablet cores, (**b**) a single 3D-printed inert minitablet core with characteristic measurements.

The 3D-printed inert minitablet cores (Figure 2) were successfully prepared using FLEX80 Transparent Clear Prusament Resin and the Original Prusa SL1S SPEED SLA 3D printer. The high resolution of the printlets, which can be obtained with SLA printing, enabled the printing of inert cores which can meet the size criteria of minitablets. The printed minitablets match the designed specifications, featuring a consistent cylindrical shape with lofted edges. Post-curing with isopropyl alcohol washing and UV curing resulted in solid, defect-free structures. Visual inspection under the Keyence VHX-7000 digital microscope confirmed the uniformity and integrity of the printlets, with no major surface imperfections observed.

**Figure 3:**
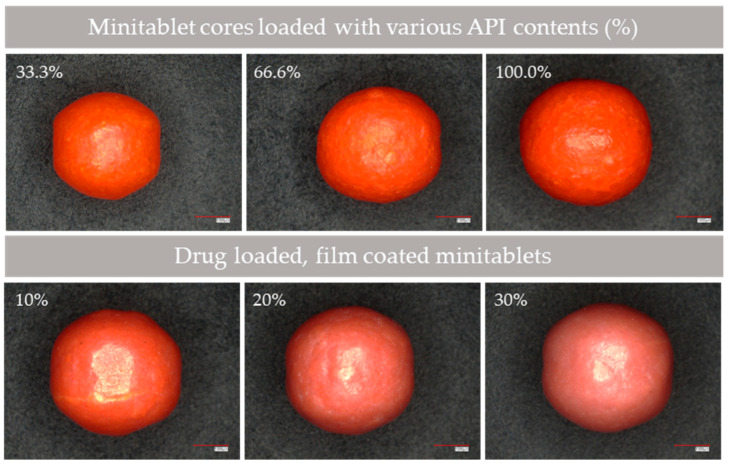
Drug-layered and film-coated minitablets. Scale bar = 1000 μm.

The fluidised bed drug layering process led to a uniform distribution of the ibuprofen sodium-containing layer onto the inert cores. A 100% increase in weight was achieved, indicating complete coverage and consistent drug loading across the batch (Figure 3). Film coating with Eudragit RS 30 dispersions was also successfully carried out. Samples taken after weight increases of 10%, 20%, and 30% confirmed the controlled application of the coating material (Figure 3), ensuring reproducibility between batches.

### 3.2. Physical Characterisation

#### 3.2.1. Mass Uniformity, Friability, and Hardness

The results of the weight uniformity test are presented in Table 3, with the test performed in accordance with Ph. Eur. 11 using 20 randomly selected units. The average weight of the 3D-printed minitablet cores was 18.56 mg. Following ibuprofen layering, the weight of the cores increased to approximately twice the initial value, with only a slight additional increase resulting from the application of the polymer film coating.

After the friability test, the inert minitablet cores exhibited no detectable mass loss, resulting in a friability value of 0%. The mean hardness of the printlets was 483.4 N (SD ± 1.35 N).

#### 3.2.2. Image Analysis

These quantitative and qualitative findings were supported by high-resolution images captured using the Keyence VHX-7000 digital microscope. Image analysis of multiple minitablets (*n* = 75) showed that the units retained their structural uniformity after drug layering and coating. The surfaces remained generally smooth; however, a slight increase in surface roughness was observed with higher film weight gains. No cracks or visible defects were detected, indicating good adhesion of the film coatings to the minitablet surfaces.

Image analysis measurements revealed that, with each applied layer, the surface area of the minitablets increased, accompanied by a corresponding upward trend in perimeter values. Both the maximum and minimum Feret diameters increased, indicating uniform growth in overall dimensions. The difference between the maximum and minimum Feret diameters remained approximately constant, confirming that the minitablets preserved their isotropic geometry and that dimensional growth occurred in all directions. Notably, the application of the 1:1 core-API layer resulted in a markedly greater increase in the measured parameters compared with subsequent layer applications.

A summary of all characterisation results is presented in Table 3.

### 3.3. Raman Microscopic Analysis

**Figure 4:**
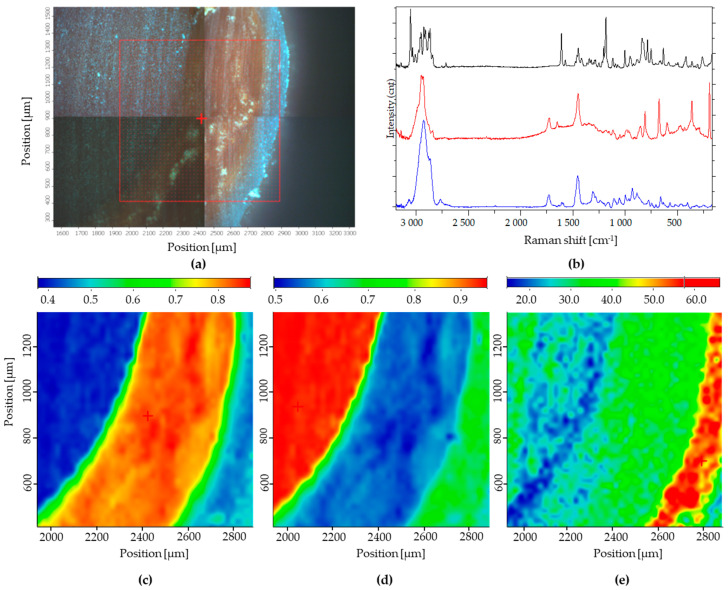
Raman spectral profiles and chemical distribution maps of the minitablet cross-section (Thermo Fisher DXR;780 nm excitation): (**a**) Microscopic image of the measured surface and the Raman mapping region (red square with grid overlay); (**b**) Raman spectra of the individual components (from top to bottom: API layer, film layer; inert core). Spectra are displayed with a constant vertical offset along the y-axis for clarity; the intensity is therefore shown in relative units (a.u). Raman chemical maps showing the spatial distribution of the API layer (**c**), 3D-printed inert minitablet core (**d**), film-forming shell (**e**) across the cross-section based on full spectral or characteristic peak profiling.

Raman maps of the minitablet are presented in Figure 4. Reference spectra of individual ingredients (Figure 4b) were applied for spectral profiling, except for film coating, where only a characteristic peak at 811 cm^−1^ was used for mapping, as the compound exhibited significantly lower overall Raman signal intensity relative to the other ingredients. For clarity, the individual spectra shown in Figure 4b were vertically offset along the y-axis; the intensity scale therefore reflects relative signal levels, and no baseline correction or smoothing was applied. In each Raman chemical image, red indicates a high concentration of the respective component, while yellow and green correspond to medium and low levels, respectively. Blue regions represent areas where the characteristic signal of the given component was not detected.

Raman mapping qualitatively confirmed the macroscopic core–shell structure of the layered minitablets. Characteristic API bands were predominantly localised in the inner shell, while no API-related signals were detected in the inert core or the outer film layer. The core spectrum was clearly distinguishable and confined to the central region, indicating that no Raman signal of the active ingredient was observed within the core under the applied experimental conditions. The film-forming polymer appeared as a continuous outer band without visible discontinuities at the applied spatial resolution (10× objective, 22 µm step size), which was sufficient to resolve the overall geometry of the core–shell-coating structure.

### 3.4. Study of Drug Release

**Figure 5:**
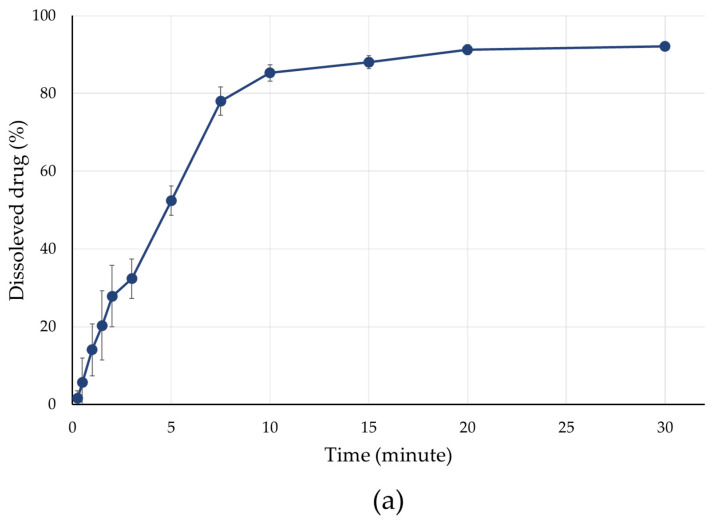

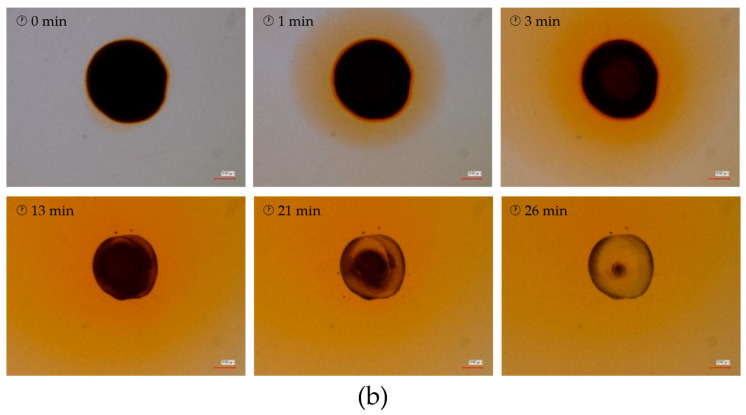
Active ingredient release. (**a**) Time-dependent release of API, kinetics study over 30 min, (**b**) Visualisation of API dissolution behaviour from the 3D printed inert minitablet carrier. Scale bar = 1000 μm.

The in vitro dissolution profiles revealed that the presence and thickness of the film coating strongly influenced drug release. Uncoated minitablets showed rapid release, with over 85% of the ibuprofen sodium content within the first 10 min, as shown in Figure 5a.

**Figure 6:**
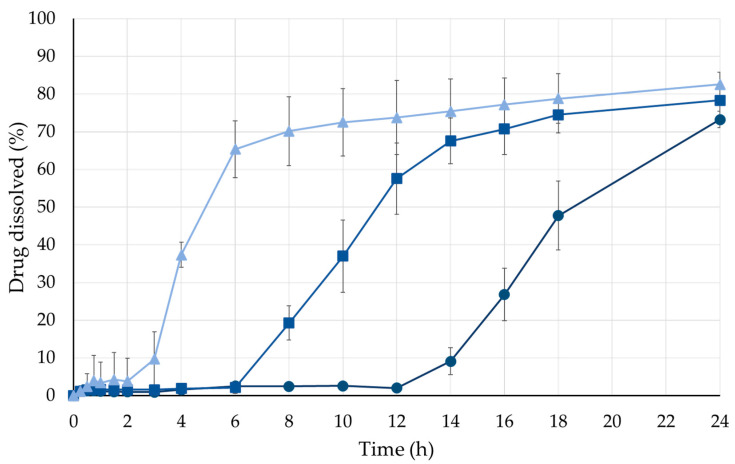
Influence of film coating thickness on ibuprofen sodium release from 3D-printed inert cores (▲: 10%; ■: 20%; ●: 30%—weight gain).

Tablets coated to a 10% weight gain showed a moderate slowdown in release, reaching 70% dissolution within approximately 8 h. Further increases in coating thickness to 20% and 30% resulted in longer release times, with 80% of the drug released after approximately 16 and 24 h (Figure 6).

These results confirmed that the Eudragit RS 30D-based coating effectively functioned as a release-modifying barrier. UV–VIS spectrophotometric analysis at 222 nm provided consistent and reliable concentration measurements, with the average standard deviations remaining below 5%, which highlights the reproducibility of the drug release testing.

**Figure 7:**
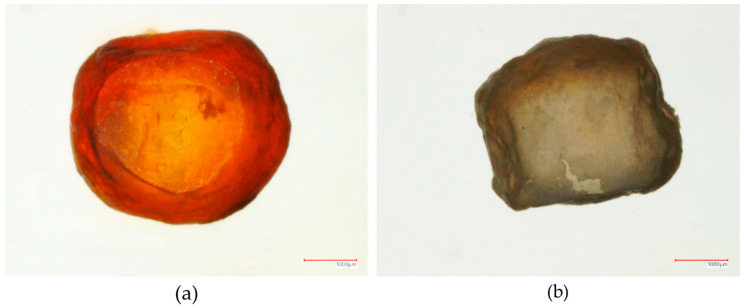
Illustration of the core during dissolution. (**a**) Before dissolution: The inert core with the drug layer distributed within the polymer shell, (**b**) After dissolution: The reconstituted inert core remaining within an intact polymer shell.

As illustrated in Figure 7, following the dissolution of the drug layer and the release of the API, the inert core becomes reconstituted within the outer polymer shell.

### 3.5. Kinetic Analysis of Dissolution Data

The drug release profiles of minitablets coated with Eudragit RS were successfully fitted to both Higuchi and Korsmeyer-Peppas models. In both cases, a clear trend was observed, as shown in Table 4: the release rate constant (*k_H_* and *k_KP_*) decreased as the polymer coating thickness increased, while thicker coatings resulted in longer lag times.

## 4. Discussion

Although many recent publications on pharmaceutical 3D printing focus on transdermal or subcutaneous drug delivery systems, it should not be overlooked that oral administration remains the most widely used and preferred route of drug delivery among patients [51,52,53,54]. Furthermore, the only FDA-approved 3D-printed pharmaceutical formulation is an orodispersible tablet (Spritam^®^; Aprecia Pharmaceuticals, Blue Ash, OH, USA) that is administered orally [55,56]. Therefore, the investigation of 3D printing technologies for oral dosage forms remains highly relevant, particularly considering the continuous technological advancements in this area [57]. Within this framework, stereolithography represents a particularly promising approach due to its nanometer-scale control over printed layers, enabling the production of dosage forms with highly defined geometries while maintaining relatively short production times. Such precision offers unique opportunities for tailoring drug release profiles and dosage form architecture. Light-curing 3D printing techniques, including SLA, rely on liquid resins composed of finely tuned mixtures of versatile components, such as monomeric or oligomeric precursors, photo initiators, light absorbers, and various additives designed to enhance processability. From a technological perspective, this compositional flexibility provides substantial potential to influence both process parameters and the critical quality attributes of the resulting dosage forms [58]. However, this advantage is accompanied by a significant limitation, as the pharmaceutical application of such resins is currently constrained by the limited availability of pharmaceutical-grade, biocompatible components. In this context, the FLEX 80 resin used in the present work serves as a model material composed of typical additive manufacturing ingredients, including acrylate–diacrylate systems and phosphinate-based components [59]. While this resin does not qualify as a pharmacopoeian excipient, it is suitable for proof-of-concept studies and the exploration of formulation and technological aspects relevant to future drug development. Despite the fact that the resins currently employed do not yet meet the criteria of the Pharmacopoeia, pioneering work by Wang et al. reported the successful printing of modified-release dosage forms containing non-steroidal anti-inflammatory drugs (NSAIDs) using SLA technology [60]. Subsequently, Madžarević et al. optimised resin formulations by increasing the proportion of biocompatible components, such as PEG 700 and riboflavin, thereby improving their suitability for pharmaceutical applications [61]. Furthermore, in vivo studies conducted in rats in 2023 provided additional evidence supporting the safe utilisation of SLA-fabricated oral dosage forms [62]. Nevertheless, ongoing research efforts continue to expand the range of biocompatible and biodegradable resins available for light-based 3D printing technologies. These developments may ultimately lead to materials that reach a level of maturity sufficient for their official recognition as pharmacopoeian excipients.

The findings of this study align well with the initial objectives, as SLA-type 3D printing proved to be a flexible technique that can be effectively used to fabricate inert minitablet cores suitable for multiparticulate drug delivery systems. As the average mass of all various samples (Table 3) was below 80 mg, a deviation limit of ±10% was applicable in accordance with the Ph. Eur. uniformity of mass requirements. Based on the measurements, the observed deviations were substantially lower than this acceptance limit. Although the standard deviation increased during the layering and film coating processes compared to that of the initial cores, it remained well below the limits defined by the Ph. Eur. criteria. Tablet friability is required to remain below 1% (Ph. Eur.). In the present study, the minitablet cores exhibited a friability of approximately 0%, with no detectable damage after testing. Their high mechanical strength and favourable geometry therefore support their suitability for fluidised bed processing. As discussed by Zaid, in addition to adequate tablet mechanical strength, geometry plays a crucial role during the coating process [63]. Using SLA 3D printing, it was possible to design and manufacture starting cores with sufficient mechanical strength and appropriate geometrical parameters for subsequent drug layering in a fluidised bed system, since insufficient mechanical strength would lead to particle fracture during movement in the fluidised bed, resulting in surface damage and preventing the formation of a uniform layer on the minitablet surfaces. Inappropriate geometry likewise adversely affects coating quality; however, chipping and edge erosion, as reported in the literature for unsuitable tablet shapes, were not observed during the drug-layering experiments. Adequate adhesion of the coating to the substrate surface is also of critical importance; therefore, HPMC was employed as the binder for the layering process, as it is widely reported in the literature for coating various active pharmaceutical ingredients [30,64]. Although drug layering is generally performed onto spherical starter cores, exceptions do exist, including commercially available coated tablets in which the active pharmaceutical ingredient, such as sirolimus, is layered onto an inert, biconvex tablet core containing only excipients [65].

The ability to customise and tailor minitablet dimensions through digital design, without altering hardware components, further highlights a key advantage of additive manufacturing over traditional production techniques, particularly for paediatric and geriatric applications, where flexibility and patient-specific adjustments are crucial.

Microscopic analyses and active ingredient content assays confirmed that the drug was successfully deposited on the surface of the resin-based minitablets. The application of fluidised bed technology for drug-layering yielded consistent and predictable results, further reinforcing the compatibility of the printed cores with well-established pharmaceutical unit operations. Ibuprofen sodium was successfully layered onto the surface of the cores, resulting in reliable endpoint weight gains. This step effectively transformed the inert core into drug-loaded minitablets, supporting the concept that 3D-printed cores can serve as viable drug carriers in multi-particulate systems as alternatives to the currently used inert cores.

Raman chemical imaging has previously been applied to characterise coated drug delivery systems [66,67,68]. The present findings further support its applicability to layered minitablets with 3D-printed cores. The spatial confinement of the API to the shell layer and its absence from the inert core demonstrate the successful formation of the intended core–shell structure. Based on the characteristic peak assigned to the film-forming layer, the coating appears as a continuous band along the outer surface of the minitablet in the mapped region, without obvious discontinuities or ‘holes’ at the spatial resolution of the present experiment, which is consistent with earlier Raman imaging work visualising coating homogeneity in core–shell systems [69]. Owing to the tens-of-micrometre spatial resolution, the method allows assessment of overall layer geometry but not fine sub-layer concentration gradients.

Furthermore, the film coating process using Eudragit RS 30D demonstrated the ability to modulate the drug release profile, thereby achieving controlled release. A direct relationship was observed between coating thickness and the rate of ibuprofen sodium release, which supports the hypothesis that the coating acts as a functional barrier to delay drug diffusion. This adjustability is particularly important for designing release profiles for different therapeutic indications. The coatings maintained their integrity without visual defects, indicating good film-forming characteristics and adhesion to the drug-layered substrates.

The observed trends in both Higuchi and Korsmeyer-Peppas models indicate that the water-insoluble, but permeable, Eudragit RS coating exerts a clear membrane-controlled effect on drug release. While the release rate constants decreased moderately with increasing coating thickness, the lag time increased several-fold, highlighting that the initial induction phase is highly sensitive to the thickness of the polymer layer. This behaviour is characteristic of membrane-controlled systems. The diffusional exponent obtained from the Korsmeyer-Peppas model remained close to 0.5 across all coating levels, suggesting that Fickian diffusion dominates, and anomalous transport plays only a minor role in the process. The combination of prolonged lag time and a relatively modest decrease in *k* confirms that the Eudragit RS coating functions as a true barrier, delaying the onset of drug release while allowing controlled diffusion once the induction period is overcome. Overall, these findings demonstrate that adjusting the coating thickness provides a straightforward strategy to modulate the induction period and release rate, which could be exploited to tailor the therapeutic treatment of coated minitablets.

The implications of these results are significant: a hybrid manufacturing approach that integrates digital design, 3D printing, and traditional pharmaceutical techniques can successfully produce customised multiparticulate dosage forms. This opens new possibilities for developing patient-centric treatments with greater dosing precision and improved compliance. The adaptability of this system also makes it an attractive candidate for therapies requiring modified release or combination dosing, especially in managing chronic conditions.

This combined platform supports future research aspects aiming at the development of multiparticulate formulations initiated with a 3D printing unit operation. Further studies should focus on scalability, long-term stability, regulatory aspects, and in vivo performance to evaluate and validate the broader pharmaceutical applicability of additive manufacturing technologies.

## 5. Conclusions

This study demonstrated that SLA 3D printing using a UV-curable resin can effectively manufacture inert minitablet cores suitable for multi-particulate drug delivery systems. The printed tablets showed consistent dimensions, mechanical strength, and surface quality, making them ideal carriers for further pharmaceutical processing.

Drug-layering using a fluidised bed method—an established and widely applied technology in pharmaceutical manufacturing—was successfully applied to these 3D-printed cores, resulting in uniform application of ibuprofen sodium. The subsequent film coating with Eudragit RS 30D enabled precise control over drug release, with thicker coatings significantly delaying dissolution.

These findings support the combination of advanced digital manufacturing techniques with traditional pharmaceutical processes. The approach offers flexibility in design, reproducibility in production, and potential for personalised therapy, which benefits patient groups with special needs, such as children or the elderly.

At the same time, a key limitation of the broader pharmaceutical application of SLA printing remains the relatively narrow range of biocompatible photopolymer resins currently available. Although previously introduced biocompatible materials, primarily developed for surgical and dental applications, show promise, the development of novel, pharmaceutically suitable resins will be essential for expanding the use of additive manufacturing technologies in oral dosage form development.

Overall, this study demonstrates the potential of 3D printing as a transformative technology for advancing next-generation drug delivery systems, especially in personalised medicine. Confirming the viability of SLA 3D-printed minitablets as platforms for controlled-release, multiparticulate formulations demonstrates how innovative manufacturing can be successfully integrated with traditional pharmaceutical processes, which represents a significant advancement in the field of drug delivery systems.

## Figures and Tables

**Table 1:** Parameters for fluidised bed processing.

Manufacturing Parameter	Drug Layering	Film Coating
Fill Weight (g)	75.0	100.0
Nozzle Diameter (mm)	0.8	0.8
Inlet Air Temperature (°C)	50.0	45
Outlet Air Temperature (°C)	31–35	37–40
Spray Air Pressure (bar)	0.8	0.8
Fluid Air Flow Rate (m^3^/hour)	60	60
Feed Rate (g/min)	2–4	3
Drying Temperature (°C)	50	45
Drying Time (min)	10	10

**Table 2:** Composition of the dispersion used for film coating.

Component	Function	*w*/*w%*
Eudragit RS 30 D	Film-forming polymer	33.30
TEC	Plasticizer	2.00
Talc	Anti-adhesion agent	7.50
Deionised Water	Dispersion medium	57.20

**Table 3:** Physical characterisation of 3D-printed uncoated, drug-layered and coated minitablets.

Sample		Weight (mg) (*n* = 20; *avg.* ± *SD*)	Image Analysis (*n* = 75; *avg.* ± *SD*)			
			Area (mm^2^)	Perimeter (mm)	Max. Feret (mm)	Min. Feret (mm)
Inert Core		18.56 ± 0.648	8.06 ± 0.371	10.98 ± 0.337	3.43 ± 0.121	3.08 ± 0.082
Drug-Layered Minitablet		36.25 ± 1.581	11.93 ± 0.553	14.34 ± 1.013	4.16 ± 0.157	3.76 ± 0.105
Coated Minitablet (Weight Gain; %)	10	40.06 ± 1.604	12.59 ± 0.480	15.73 ± 1.590	4.30 ± 0.156	3.85 ± 0.093
	20	41.89 ± 1.609	13.20 ± 0.536	15.91 ± 1.391	4.39 ± 0.189	3.95 ± 0.095
	30	44.78 ± 1.971	13.75 ± 0.614	15.07 ± 0.739	4.42 ± 0.145	4.05 ± 0.105

**Table 4:** Model fitting of the ibuprofen sodium release from polymer coated minitablets.

**Model**		**Coating Level (Weight Gain; %)**		
**Higuchi**		**10**	**20**	**30**
Release rate constant (%/h^0.5^)		22.10	21.13	19.50
t_lag_ (h)		2.60	6.52	12.90
Coefficient of determination		0.9754	0.9867	0.9555
**Model**		**Coating Level (Weight Gain; %)**		
**Korsmeyer-Peppas**		**10**	**20**	**30**
k (1/h*^n^*)		32.12	23.53	18.52
t_lag_ (h)		2.79	6.95	13.90
*n*		0.56	0.52	0.55
Coefficient of determination		0.9028	0.9757	0.9538

## Data Availability

The original contributions presented in this study are included in the article. Further inquiries can be directed to the corresponding author.

## Abbreviations

The following abbreviations are used in this manuscript:

3D	Three-dimensional
API	Active pharmaceutical ingredient
FDM	Fused deposition modelling
HPMC	Hydroxypropyl methylcellulose
m/m%	Mass/mass per cent
MUPS	Multi-unit particulate systems
ODMTs	Orodispersible minitablets
Ph. Eur.	European Pharmacopoeia
SLA	Stereolithography
TEC	Triethyl citrate
UV	Ultraviolet

## References

[1] Limenh L.W., Tessema T.A., Simegn W., Ayenew W., Bayleyegn Z.W., Sendekie A.K., Chanie G.S., Fenta E.T., Beyna A.T., Kasahun A.E. (2024). Patients’ Preference for Pharmaceutical Dosage Forms: Does It Affect Medication Adherence? A Cross-Sectional Study in Community Pharmacies. Patient Prefer. Adher..

[2] Al-Hashimi N., Begg N., Alany R.G., Hassanin H., Elshaer A. (2018). Oral Modified Release Multiple-Unit Particulate Systems: Compressed Pellets, Microparticles and Nanoparticles. Pharmaceutics.

[3] Kallai-Szabo N., Farkas D., Lengyel M., Basa B., Fleck C., Antal I. (2024). Microparticles and multi-unit systems for advanced drug delivery. Eur. J. Pharm. Sci..

[4] Münch J., Sessler I., Bosse H.M., Wargenau M., Dreesen J.D., Loforese G., Webb N.J.A., Sivasubramanian R., Reidemeister S., Lustenberger P. (2023). Evaluating the Acceptability, Swallowability, and Palatability of Film-Coated Mini-Tablet Formulation in Young Children: Results from an Open-Label, Single-Dose, Cross-Over Study. Pharmaceutics.

[5] Priese F., Wiegel D., Funaro C., Mondelli G., Wolf B., Ciobanu G. (2023). Comparison of Mini-Tablets and Pellets as Multiparticulate Drug Delivery Systems for Controlled Drug Release. Coatings.

[6] Agrawal S., Fernandes J., Shaikh F., Patel V. (2022). Quality aspects in the development of pelletized dosage forms. Heliyon.

[7] Beker L.T., Fink R.J., Shamsa F.H., Chaney H.R., Kluft J., Evans E., Schidlow D.V. (1994). Comparison of Weight-Based Dosages of Enteric-Coated Microtablet Enzyme Preparations in Patients with Cystic-Fibrosis. J. Pediatr. Gastroenterol. Nutr..

[8] de Mey C., Dimitrova V., Lennartz P., Wangemann M. (2012). Bioequivalence of a Novel Minitablet Formulation of Levetiracetam. Arzneimittelforschung.

[9] Hejduk A., Lulek J. (2022). Dispensing of minitablets-Has the problem been resolved?. Int. J. Pharm..

[10] Lura V., Lura A., Breitkreutz J., Klingmann V. (2025). The revival of the mini-tablets: Recent advancements, classifications and expectations for the future. Eur. J. Pharm. Biopharm..

[11] Turk M., Sibanc R., Dreu R., Frankiewicz M., Sznitowska M. (2021). Assessment of Mini-Tablets Coating Uniformity as a Function of Fluid Bed Coater Inlet Conditions. Pharmaceutics.

[12] De Brabander C., Vervaet C., Remon J.P. (2003). Development and evaluation of sustained release mini-matrices prepared via hot melt extrusion. J. Control. Release.

[13] Poller B., Strachan C., Broadbent R., Walker G.F. (2017). A minitablet formulation made from electrospun nanofibers. Eur. J. Pharm. Biopharm..

[14] Verhoeven E., Siepmann F., De Beer T.R.M., Van Loo D., Van den Mooter G., Remon J.P., Siepmann J., Vervaet C. (2009). Modeling drug release from hot-melt extruded mini-matrices with constant and non-constant diffusivities. Eur. J. Pharm. Biopharm..

[15] Wang S., Chen X., Han X., Hong X., Li X., Zhang H., Li M., Wang Z., Zheng A. (2023). A Review of 3D Printing Technology in Pharmaceutics: Technology and Applications, Now and Future. Pharmaceutics.

[16] Krause J., Müller L., Sarwinska D., Seidlitz A., Sznitowska M., Weitschies W. (2021). 3D Printing of Mini Tablets for Pediatric Use. Pharmaceuticals.

[17] Kim Y.J., Choi Y.R., Kang J.H., Park Y.S., Kim D.W., Park C.W. (2024). Geometry-Driven Fabrication of Mini-Tablets via 3D Printing: Correlating Release Kinetics with Polyhedral Shapes. Pharmaceutics.

[18] Hu J.Y., Fitaihi R., Abukhamees S., Abdelhakim H.E. (2023). Formulation and Characterisation of Carbamazepine Orodispersible 3D-Printed Mini-Tablets for Paediatric Use. Pharmaceutics.

[19] Oldfield L.R., Fischer B., Auel T., Seidlitz A. (2026). Development of a 3D printed multiple unit particle system (MUPS) containing metoprolol succinate. Eur. J. Pharm. Sci..

[20] Trenfield S.J., Awad A., Madla C.M., Hatton G.B., Firth J., Goyanes A., Gaisford S., Basit A.W. (2019). Shaping the future: Recent advances of 3D printing in drug delivery and healthcare. Expert. Opin. Drug Deliv..

[21] Zhang J., Feng X., Patil H., Tiwari R.V., Repka M.A. (2017). Coupling 3D printing with hot-melt extrusion to produce controlled-release tablets. Int. J. Pharm..

[22] Mora-Castano G., Millan-Jimenez M., Linares V., Caraballo I. (2022). Assessment of the Extrusion Process and Printability of Suspension-Type Drug-Loaded Affinisol(TM) Filaments for 3D Printing. Pharmaceutics.

[23] Awad A., Fina F., Trenfield S.J., Patel P., Goyanes A., Gaisford S., Basit A.W. (2019). 3D Printed Pellets (Miniprintlets): A Novel, Multi-Drug, Controlled Release Platform Technology. Pharmaceutics.

[24] Anabousi S., Naseef H., Qurt M., AbuKhalil A., Rabba A. (2025). Fast disintegrating pellets: Formulation and evaluation. F1000Res.

[25] Kallai-Szabo N., Lengyel M., Farkas D., Barna A.T., Fleck C., Basa B., Antal I. (2022). Review on Starter Pellets: Inert and Functional Cores. Pharmaceutics.

[26] Kadry H., Wadnap S., Xu C., Ahsan F. (2019). Digital light processing (DLP) 3D-printing technology and photoreactive polymers in fabrication of modified-release tablets. Eur. J. Pharm. Sci..

[27] Norman J., Madurawe R.D., Moore C.M.V., Khan M.A., Khairuzzaman A. (2017). A new chapter in pharmaceutical manufacturing: 3D-printed drug products. Adv. Drug Deliver. Rev..

[28] Alhnan M.A., Okwuosa T.C., Sadia M., Wan K.W., Ahmed W., Arafat B. (2016). Emergence of 3D Printed Dosage Forms: Opportunities and Challenges. Pharm. Res..

[29] Kállai N., Luhn O., Dredán J., Kovács K., Lengyel M., Antal I. (2010). Evaluation of Drug Release from Coated Pellets Based on Isomalt, Sugar, and Microcrystalline Cellulose Inert Cores. AAPS PharmSciTech.

[30] Vlahovic K., Lengyel M., Fleck C., Kallai-Szabo N., Balogh E., Laki A.J., Antal I. (2025). Microenvironmental pH-Modulated Dissolution of Albendazole Layered on Tartaric Acid Starter Pellet Cores. Pharmaceutics.

[31] Lopes C.M., Lobo J.M.S., Costa P., Pinto J.F. (2006). Directly compressed mini matrix tablets containing ibuprofen: Preparation and evaluation of sustained release. Drug Dev. Ind. Pharm..

[32] Abbaspour M.R., Sadeghi F., Garekani H.A. (2005). Preparation and characterization of ibuprofen pellets based on Eudragit RS PO and RL PO or their combination. Int. J. Pharm..

[33] Borbás B., Kohod Z., Kállai-Szabó N., Basa B., Lengyel M., Zelkó R., Antal I. (2025). Evaluation of 3D-Printed Balls with Photopolymer Resin as Grinding Medium Used to Alternatively Reduce Warmup During Dry Milling. Polymers.

[34] Basa B., Jakab G., Kallai-Szabo N., Borbas B., Fulop V., Balogh E., Antal I. (2021). Evaluation of Biodegradable PVA-Based 3D Printed Carriers during Dissolution. Materials.

[35] Goyanes A., Martinez P.R., Buanz A., Basit A.W., Gaisford S. (2015). Effect of geometry on drug release from 3D printed tablets. Int. J. Pharm..

[36] Oldfield L.R., Mentrup A.F.C., Klinken-Uth S., Auel T., Seidlitz A. (2024). From design to 3D printing: A proof-of-concept study for multiple unit particle systems (MUPS) printed by dual extrusion fused filament fabrication. Int. J. Pharm. X.

[37] Grygier D., Kurzawa A., Stachowicz M., Krawiec K., Stepczak M., Roszak M., Kazimierczak M., Aniszewska D., Pyka D. (2024). Investigations into the Material Characteristics of Selected Plastics Manufactured Using SLA-Type Additive Methods. Polymers.

[38] Wang S.H., Ma Y.B., Deng Z.C., Zhang K., Dai S. (2020). Implementation of an elastoplastic constitutive model for 3D-printed materials fabricated by stereolithography. Addit. Manuf..

[39] Yang S.T., Vansavage G., Weiss J., Ghebresellassie I. (1992). The Effect of Spray Mode and Chamber Geometry of Fluid-Bed Coating Equipment and Other Parameters on an Aqueous-Based Ethylcellulose Coating. Int. J. Pharm..

[40] Chen T., Li J., Chen T., Sun C.C., Zheng Y. (2017). Tablets of multi-unit pellet system for controlled drug delivery. J. Control. Release.

[41] Debunne A., Vervaet C., Mangelings D., Remon J.P. (2004). Compaction of enteric-coated pellets: Influence of formulation and process parameters on tablet properties and in vivo evaluation. Eur. J. Pharm. Sci..

[42] Council of Europe (2023). European Pharmacopoeia.

[43] Meruva S., Singaraju A.B., Vinjamuri B.P., Ternik R., Stagner W.C. (2024). Current State of Minitablet Product Design: A Review. J. Pharm. Sci..

[44] Aleksovski A., Dreu R., Gasperlin M., Planinsek O. (2015). Mini-tablets: A contemporary system for oral drug delivery in targeted patient groups. Expert. Opin. Drug Deliv..

[45] Czajkowska M., Sznitowska M., Kleinebudde P. (2015). Determination of coating thickness of minitablets and pellets by dynamic image analysis. Int. J. Pharm..

[46] Kandpal L.M., Cho B.K., Tewari J., Gopinathan N. (2018). Raman spectral imaging technique for API detection in pharmaceutical microtablets. Sens. Actuators B Chem..

[47] Krotova S.Y., Chirgin A.V., Ilin A.E. (2019). Analysis of software products for processing the results of spectroscopy. Int. J. Mech. Eng. Technol..

[48] Rabbat C., Pinna A., Andres Y., Villot A., Awad S. (2023). Adsorption of ibuprofen from aqueous solution onto a raw and steam-activated biochar derived from recycled textiles insulation panels at end-of-life: Kinetic, isotherm and fixed-bed experiments. J. Water Process Eng..

[49] Mehta R., Chawla A., Sharma P., Pawar P. (2013). Formulation and in vitro evaluation of Eudragit S-100 coated naproxen matrix tablets for colon-targeted drug delivery system. J. Adv. Pharm. Technol. Res..

[50] Dash S., Murthy P.N., Nath L., Chowdhury P. (2010). Kinetic modeling on drug release from controlled drug delivery systems. Acta Pol. Pharm..

[51] Zhu H., Kuang H., Huang X., Li X., Zhao R., Shang G., Wang Z., Liao Y., He J., Li D. (2025). 3D printing of drug delivery systems enhanced with micro/nano-technology. Adv. Drug Deliv. Rev..

[52] Lau K., Tran H.A., Tan R., Kumeria T., Prasad A.A., Tan R.P., Lim K.S. (2025). Advancements in 3D-printing strategies towards developing effective implantable drug delivery systems: Recent applications and opportunities. Adv. Drug Deliv. Rev..

[53] Correia A., Agostinho Cordeiro M., Mendes M., Marques Ribeiro M., Mascarenhas-Melo F., Vitorino C. (2026). Additive manufacturing of microneedles: A quality by design approach to clinical translation. Int. J. Pharm..

[54] Fu X., Sun Z., Gu J., Liu R., Ma M., Ma X., Ho Y.P., Zhang X., Zhang Y. (2026). 3D-printed barbed microneedle electrodes for biosensing and drug delivery in wound management. Microsyst. Nanoeng..

[55] Saleh-Bey-Kinj Z., Heller Y., Socratous G., Christodoulou P. (2025). 3D Printing in Oral Drug Delivery: Technologies, Clinical Applications and Future Perspectives in Precision Medicine. Pharmaceuticals.

[56] Wang Z., Han X., Chen R., Li J., Gao J., Zhang H., Liu N., Gao X., Zheng A. (2021). Innovative color jet 3D printing of levetiracetam personalized paediatric preparations. Asian J. Pharm. Sci..

[57] Pan S., Ding S., Zhou X., Zheng N., Zheng M., Wang J., Yang Q., Yang G. (2024). 3D-printed dosage forms for oral administration: A review. Drug Deliv. Transl. Res..

[58] Deshmane S., Kendre P., Mahajan H., Jain S. (2021). Stereolithography 3D printing technology in pharmaceuticals: A review. Drug Dev. Ind. Pharm..

[59] ITW Performance Polymers (2023). Prusament Resin Flex80 Safety Datasheet.

[60] Wang J., Goyanes A., Gaisford S., Basit A.W. (2016). Stereolithographic (SLA) 3D printing of oral modified-release dosage forms. Int. J. Pharm..

[61] Madzarevic M., Medarevic D., Vulovic A., Sustersic T., Djuris J., Filipovic N., Ibric S. (2019). Optimization and Prediction of Ibuprofen Release from 3D DLP Printlets Using Artificial Neural Networks. Pharmaceutics.

[62] Mosley-Kellum K., Bagde A., Spencer S., Dev S., Singh M. (2024). Correction: Development of 3D DLP Printed Sustained Release Ibuprofen Tablets and Their Pharmacokinetic Evaluation in Rats. AAPS PharmSciTech.

[63] Zaid A.N. (2020). A Comprehensive Review on Pharmaceutical Film Coating: Past, Present, and Future. Drug Des. Devel. Ther..

[64] Peterfi O., Kallai-Szabo N., Demeter K.R., Barna A.T., Antal I., Szabo E., Sipos E., Nagy Z.K., Galata D.L. (2025). Artificial intelligence-aided endoscopic in-line particle size analysis during the pellet layering process. J. Pharm. Anal..

[65] Pfizer (2025). Rapamune 1 Mg Coated Tablet Summary of Product Characteristics.

[66] Koutentaki G., Krysa P., Trunov D., Pekarek T., Pislova M., Soos M. (2023). 3D Raman mapping as an analytical tool for investigating the coatings of coated drug particles. J. Pharm. Anal..

[67] Zakowiecki D., Frankiewicz M., Hess T., Cal K., Gajda M., Dabrowska J., Kubiak B., Paszkowska J., Wiater M., Hoc D. (2021). Development of a Biphasic-Release Multiple-Unit Pellet System with Diclofenac Sodium Using Novel Calcium Phosphate-Based Starter Pellets. Pharmaceutics.

[68] Galata D.L., Peterfi O., Ficzere M., Szabo-Szocs B., Szabo E., Nagy Z.K. (2025). The current state-of-the art in pharmaceutical continuous film coating—A review. Int. J. Pharm..

[69] Bannerman A., Williams R.L., Cox S.C., Grover L.M. (2016). Visualising phase change in a brushite-based calcium phosphate ceramic. Sci. Rep..

